# Autoimmune encephalitis in a South Asian population

**DOI:** 10.1186/s12883-021-02232-6

**Published:** 2021-05-19

**Authors:** Nilanka Wickramasinghe, Dhanushka Dasanayake, Neelika Malavige, Rajiva de Silva, Thashi Chang

**Affiliations:** 1 Department of Physiology, University of Colombo, Colombo, Sri Lanka; 2 Department of Immunology, Medical Research Institute, Colombo, Sri Lanka; 3 Department of Immunology and Molecular Medicine, University of Sri Jayawardenapura, Nugegoda, Sri Lanka; 4 Department of Clinical Medicine, Faculty of Medicine, University of Colombo, 25, Kynsey Road, Colombo, 00800 Sri Lanka

**Keywords:** Autoimmune, Encephalitis, Sri Lanka, NMDAR, Limbic, Antibody

## Abstract

**Background:**

Autoimmune encephalitis (AE) is now considered a main, potentially curable cause of encephalitis, but remains conspicuously underreported from South Asia. We studied the clinical characteristics in relation to their antibody status and outcomes of patients presenting with AE in Sri Lanka.

**Methods:**

Patients admitting to government hospitals who were clinically suspected of AE by an on-site neurologist were prospectively recruited over a period of 12 months. Sera and cerebrospinal fluid were tested for NMDAR, AMPAR1, AMPAR2, LGI1, CASPR2, GABARB1/B2 antibodies (Ab) using commercial cell-based assays. Demographic, clinical and laboratory data were compiled into an investigator-administered proforma. Patients were reviewed at 1 year follow up either in person or via telephone.

**Results:**

One-hundred and forty-two patients from 21 of 25 districts in Sri Lanka (median age = 20.5 years; range 1–86 years; females = 61.3%) were recruited. Of them, 65 (45.8%; median age = 19 years; range 1–86 years; females = 64.6%) fulfilled diagnostic criteria for probable NMDAR-antibody encephalitis (NMDARE) and 6 (4.2%; median age = 44 years; range 28–71 years; females = 83.3%) limbic encephalitis (LE). Abnormal behaviour (95.3%), seizures (81.5%) and movement disorders (69.2%) were the most frequent clinical manifestations of probable NMDARE. NMDAR-antibodies were detectable in 29 (44.6%) and not detectable in 36 in CSF of probable-NMDARE patients. Abnormal EEG was more frequent (*p* = 0.003) while a worse outcome (OR = 2.78; 95% CI = 0.88–9.09) and deaths (OR = 2.38; 95% CI = 0.67–8.33) were more likely in antibody-negative than antibody-positive probable-NMDARE. Most patients with LE had amnesia (50%) and/or confusion (100%) with agitation (83.3%) and seizures (100%) but none had detectable antibodies to any of the antigens tested.

**Conclusions:**

NMDARE is the commonest type of AE among South Asians as is the case worldwide. Clinical presentations of NMDARAb-positive and NMDARAb-negative AE patients do not significantly differ but EEG may be a useful marker of an autoimmune basis for psychiatric symptoms.

## Background

Among an estimated annual incidence of approximately 5 to 8 cases of encephalitis per 100,000 persons, autoimmune encephalitis (AE) has emerged as the third most common cause after infections, mostly viral, and acute disseminated encephalomyelitis [1]. More importantly, AE associated with autoantibodies directed against neuronal cell surface/synaptic proteins have emerged as the most treatment responsive encephalitis with the greatest potential for complete recovery [2]. Among the antibody mediated encephalitides, NMDAR-antibody encephalitis (NMDARE) is the most common followed by limbic encephalitis (LE) mediated by antibodies directed against LGI1, CASPR2, AMPAR or GABA_B_R while encephalitis mediated by other antibodies are rare [2]. NMDARE is characterised by a female predominance (4:1), younger onset (median age 21 years), associated tumours (ovarian teratoma) and a multi-phenomenological syndrome that evolves over time with seizures, abnormal movements, insomnia and irritability more frequent in children, and psychosis, abnormal behaviour, dysautonomia and coma more common in adults [3, 4]. By contrast, LE is characterised by an older age of onset (> 45 years), amnesia, confusion, seizures, hyponatraemia, increased signal of medial temporal lobes on magnetic resonance imaging and variable association with tumours determined by the associated antibody [2, 3]. LGI1 antibodies account for most of the LE and is characterised by faciobrachial dystonic seizures that may predate cognitive impairment [5].

The population of South Asia accounts for about 40% of Asia’s population and about one quarter of the world’s population. However, research on AE from this region remains sparse and limited mostly to case reports or series amounting to less than 200 cases among a 2 billion population [6–9]. Sri Lanka is an island of 65,610 km^2^, situated just south of the Indian subcontinent between northern latitudes 5^o^ to 10^o^ with a population of approximately 22 million. This study aimed to determine the prevalence of neuronal cell surface/synaptic protein binding autoantibodies, characterise the clinical manifestations and audit the treatment and outcomes among patients presenting to hospital with a syndrome clinically suggestive of AE in Sri Lanka.

## Methods

All methods were performed in accordance with the relevant guidelines and regulations of the Ethics Review Committee of the Medical Research Institute, Colombo, Sri Lanka.

### Patients and samples

Consecutive patients over the age of one-year meeting diagnostic criteria for ‘possible’ AE [10] as determined by an on-site consultant neurologist were prospectively recruited from patients admitted to government hospitals over a period of 12 months, and specimens of their sera and cerebrospinal fluid (CSF) were transported to the Department of Immunology, Medical Research Institute, Colombo, for testing. Patients who were HIV-positive or who had an alternative diagnosis that could mimic encephalitis such as psychiatric illness, metabolic disorders, epilepsy, post-anoxia, vasculitis, stroke and septicaemia, and in whom lumbar puncture was contraindicated were excluded. Written informed consent was obtained from the patient, next-of-kin or guardian. Serum and CSF were obtained from all patients when these specimens were collected as part of their diagnostic work up. Demographic, clinical and laboratory data including CSF analysis, blood investigations, brain imaging and electroencephalogram (EEG) results were compiled into a proforma that was provided to the Neurology Units in government hospitals by junior medical officers of the referring Unit. Data were verified by the research team via telephone conversation with referring physicians. Patients admitted to the two apex tertiary care hospitals in Colombo (National Hospital of Sri Lanka and Lady Ridgeway Hospital for children) and their hospital records were personally examined by the research team. Patients were reviewed after discharge from hospital at 1 year follow up either in person or via telephone.

Clinical classification of patients based on established diagnostic criteria for ‘probable’ NMDARE and LE [10] were done by researchers unaware of the antibody results.

### Laboratory analyses

**Fig. 1:**
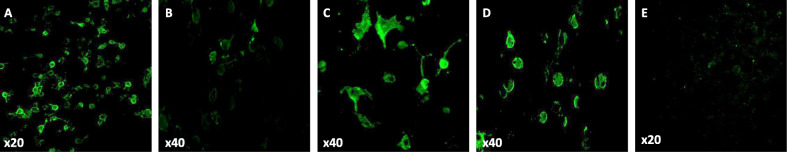
Undiluted CSF antibodies binding to NMDA-NR1 subunit-transfected HEK293T cells (Autoimmune encephalitis mosaic-6, EUROIMMUN, Lübeck, Germany) detected using fluorescein isothiocyanate-conjugated human IgG. **a** CSF positive (scored 2) × 20; **b** CSF positive (scored 1) × 40; **c** CSF positive (scored 2) × 40; **d** positive commercial control × 40; **e** CSF negative (scored 0). Immunofluorescence intensity was scored as 2 (=positive control); as 1 for low positive; and as 0 for negative. NMDAR = N-methyl-D-aspartate receptor, HEK = Human embryonic kidney

Undiluted CSF from all patients and 1:10 diluted serum from patients clinically suspected of LE, which can be negative in CSF, were assayed for autoantibodies binding to NMDAR, AMPA1 and AMPA2 receptors, LGI1, CASPR2 and GABAR_B1/B2_ using Autoimmune Encephalitis Mosaic 6 kit from EUROIMMUN, Lübeck, Germany according to the manufacturer’s instructions. The biochip slides were examined under indirect immunofluorescence microscope at × 20 and × 40 magnification by two independent assessors (TC and RdS) trained in cell-based assays and who were unaware of the clinical details of the patients. Specimens were classified as positive or negative based on the intensity of surface immunofluorescence in comparison with positive and negative controls provided in the kit (Fig. 1). The intensity was scored as 0 (=negative control), 1 (borderline positive) and 2 (=positive control). Both 1 and 2 were considered as positive.

Routine microbiological screening of CSF was performed using Gram stain, Ziehl Neelsen stain and culture on enriched culture media and when indicated, polymerase chain reaction assay for *Herpes simplex* virus.

### Data analysis

Statistical analysis was done using SPSS Statistics 26 software. Pearson’s chi-squared test was used to determine whether there is a statistically significant difference between categories compared.

## Results

A total of 142 patients from 21 of the 25 districts of Sri Lanka fulfilling criteria for ‘possible’ AE [10] were referred by neurologists for inclusion. Almost a fifth of the study population was from the district of Colombo. Patient ages ranged from 1 to 86 years (mean = 27.8; SD = 20.3) and most were female (61.3%).

**Fig. 2:**
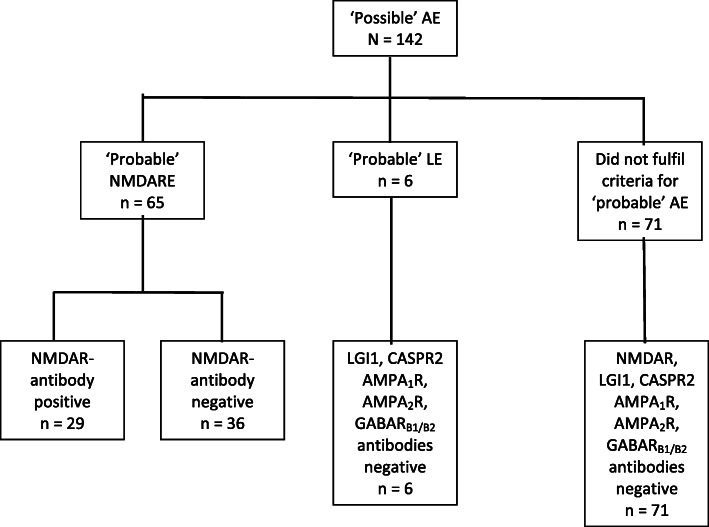
Algorithm of diagnosis of autoimmune encephalitis using a clinical approach and neuroglial surface autoantibody testing. Patients presenting with encephalitis were classified as ‘possible’ and ‘probable’ AE based on Graus criteria [10]. CSF was tested first. Serum was tested only in those without detectable antibodies in CSF. None were seropositive. AE = autoimmune encephalitis; NMDARE = N-methyl-D-aspartate receptor antibody encephalitis; LE = limbic encephalitis

Of the 142 patients with ‘possible’ AE, 71 (50%) fulfilled diagnostic criteria of ‘probable’ NMDARE or LE [10] (Fig. 2). Clinical characteristics, investigation findings, treatment and outcome of antibody-positive and antibody-negative AE patients are given in Table 1.

**Table 1:** Clinical characteristics, investigation findings, treatment and outcome of antibody-positive and antibody-negative AE patients

Characteristic	Clinically diagnosed NMDARE				Clinically diagnosed LE n (%)
	Total N (%)	NMDAR-Ab positive n (%)	NMDAR-Ab negative^a^ n (%)	NMDAR-Ab positive Vs negative ***p***-value	
Patients	65 (100)	29 (20.4)	36 (25.4)		6 (4.2)
Mean age (SD)	25.6 (20.3)	19.3 (11.9)	30.7 (24.1)	**0.01**	47.2 (17.5)
Females	42 (64.6)	22 (75.9)	20 (55.5)	0.09	5 (83.3)
**Clinical manifestations**					
Decreased level of consciousness	58 (89.2)	23 (79.3)	35 (97.2)	**0.01**	4 (66.6)
Abnormal (psychiatric) behaviour or cognitive dysfunction	62 (95.3)	27 (93.1)	35 (97.2)	0.43	6 (100)
Amnesia/working memory deficits	28 (43.0)	9 (31.0)	19 (52.8)	0.08	3 (50)
Confusion/disorientation in time, place or person	56 (86.1)	24 (82.8)	32 (88.9)	0.48	6 (100)
Visual hallucinations	19 (29.2)	9 (31.0)	10 (27.8)	0.77	1 (16.6)
Auditory hallucinations	15 (23.1)	8 (27.6)	7 (19.4)	0.44	0
Insomnia	24 (36.9)	12 (41.4)	12 (33.3)	0.50	3 (50.0)
Agitation	36 (55.4)	19 (65.5)	17 (47.2)	0.14	5 (83.3)
Catatonia	23 (35.4)	9 (31.0)	14 (38.9)	0.51	0
Obsessive thoughts or actions	13 (20.0)	6 (20.7)	7 (19.4)	0.90	0
Speech dysfunction	30 (46.1)	7 (24.1)	23 (63.9)	**0.001**	0
Seizures	53 (81.5)	25 (86.2)	28 ((77.7)	0.38	6 (100)
Focal	32 (49.2)	15 (51.7)	17 (47.2)	0.72	2 (33.3)
Generalised	34 (52.3)	15 (51.7)	19 (52.8)	0.93	4 (66.6)
Status epilepticus	18 (27.7)	9 (31.0)	9 (25)	0.59	1 (16.6)
Faciobrachial dystonic seizures	0	0	0	0	0
Movement disorders	45 (69.2)	17 (58.6)	28 (77.7)	0.09	0
Orofacial dyskinesia	30 (46.2)	11 (37.9)	19 (52.8)	0.23	0
Limb dyskinesia	25 (38.2)	10 (34.5)	15 (41.7)	0.55	0
Parkinsonism	5 (7.7)	1 (3.4)	4 (11.1)	0.25	0
Dysautonomia	16 (24.6)	6 (20.6)	10 (27.7)	0.51	0
Hyperhidrosis	4 (6.2)	2 (6.9)	2 (5.6)	0.83	0
Hypersalivation	9 (13.8)	4 (13.8)	5 (13.9)	0.99	0
Fluctuating heart rate	6 (9.2)	2 (6.9)	2 (5.6)	0.56	0
Fluctuating blood pressure	4 (6.2)	2 (6.9)	2 (5.6)	0.83	0
Central hypoventilation	0	0	0		0
Tumours	0	0	0		0
**Investigations**					
CSF protein elevated (number/of available data)	21/57 (36.8)	7/24 (29.2)	14/33 (42.2)	0.31	3/5 (60.0)
CSF pleocytosis (number/of available data)	22/58 (37.9)	10/26 (38.5)	12/32 (37.5)	0.94	5/5 (100.0)
Abnormal EEG (number/of available data)	46/56 (82.1)	14/22 (63.6)	32/34 (94.1)	**0.003**	2/4 (50.0)
Abnormal MRI (number/of available data)	13/40 (32.5)	4/16 (25.0)	9/24 (37.5)	0.41	1/3 (33.3)
**Treatment**					
Intravenous methylprednisolone	41 (63.0)	20 (68.9)	21 (58.3)	0.38	6 (100)
Intravenous immunoglobulins	34 (52.3)	17 (58.6)	17 (47.2)	0.36	2 (33.3)
Plasmapheresis	35 (53.8)	19 (65.5)	16 (44.4)	0.09	0
Rituximab	6 (9.2)	4 (13.8)	2 (5.5)	0.25	0
Mycophenolate mofetil	2 (3.0)	1 (3.4)	1 (2.7)	0.25	0
Anti-epileptic medication	37 (56.9)	16 (55.1)	21 (58.3)	0.79	4 (66.6)
**Outcome at one-year review**					
Recovery to mRS < 3 (number/of available data)	41/60 (68.3)	23/29 (79.3)	18/31 (58.0)	0.07	3/5 (60.0)
Deaths	14/60 (23.3)	4 (13.8)	10 (27.8)	0.17	2 (33.3)

^a^CSF was tested first. Serum was tested only in those without detectable antibodies in CSF. None were seropositive

Sixty-five patients (mean age = 25.6 years; SD = 20.3; 49.2% less than 18 years of age; 64.6% females) fulfilled diagnostic criteria for ‘probable’ NMDARE [10]. Abnormal behaviour (95.3%), seizures (81.5%) and movement disorders (69.2%) were the most frequent clinical manifestations while speech dysfunction (46.1%) and dysautonomia (24.6%) were less common. No ovarian or other tumours were detected in imaging studies (MRI or CT, depending on availability). EEG was the most sensitive investigation in detecting cerebral abnormality (82.1%) while MRI and CSF analysis were abnormal only in about a third of patients with probable NMDARE. EEG abnormalities comprised epileptic and/or slow wave discharges. None of the investigation findings were specific. Most patients received either one or a combination of first line immunosuppressive/immunomodulatory therapy (intravenous methyl prednisolone 1 g/d for 5 days; intravenous immunoglobulins 0.4 g/kg/d for 5 days; 5 cycles of plasmapheresis every other day) while 9.2% received second line immunotherapy (rituximab 375 mg/m^2^ × 4 doses at weekly intervals). More than half of the probable NMDARE patients required antiepileptic medication. At one-year review, 14 (23.3%) had died, 41 (68.3%) had a good outcome (mRS < 3) and 8.3% had persistent disabling deficits. Five were lost to follow up. Five of the six patients (83.3%) who received rituximab had a good outcome.

Of the 65 patients with probable NMDARE on clinical grounds, only 29 (44.6%) had NMDAR-antibodies detectable in their CSF (Fig. 1). None of the other antibodies were detected in CSF or serum. The mean age was higher, and the female predominance less marked among the antibody-negative than the antibody-positive probable NMDARE patients. Apart for decreased level of consciousness and speech dysfunction which were more frequent among antibody-negative compared to antibody-positive probable NMDARE patients, the clinical manifestations did not significantly differ among the two groups. Of the investigations, EEG abnormalities were significantly higher (*p* = 0.003) among antibody-negative than antibody-positive probable NMDARE patients. There was no difference in the immunological interventions administered to the two groups, but a poorer response to treatment (OR = 2.78; 95% CI = 0.88–9.09) and more deaths (OR = 2.38; 95% CI = 0.67–8.33) were noted among the antibody-negative than antibody-positive patients although it did not reach statistical significance.

LE was diagnosed in 6 patients (mean age = 47.2 years; range 28–71 years; 83.3% females) based on clinical features suggestive of involvement of the limbic system as previously described [10]. Most patients had amnesia (50%) and/or confusion (100%) with agitation (83.3%) and seizures (100%), but none had faciobrachial dystonic seizures. CSF pleocytosis (5 of 5) and increased protein (3 of 5) were observed in the majority, but none had typical MRI findings of increased signal in the medial temporal lobes. All patients were treated with immunotherapy resulting in a good outcome (mRS < 3) in 60%.

Microbiological screening of the patients with ‘probable’ or ‘definite’ AE did not reveal an infectious aetiology or a concurrent CNS infection.

Of the patients remaining within the initial classification of ‘possible’ AE after excluding patients who fulfilled a diagnosis of ‘probable’ AE (*n* = 71), none were positive for encephalitogenic autoantibodies. Among them, an alternative diagnosis became evident in 67.6% subsequently with evolution of the clinical syndrome and further investigations. Diagnoses included viral encephalitis (26.8%), psychiatric illness (16.9%), epilepsy (12.7%), CNS demyelination (8.5%), pyogenic meningoencephalitis, septic encephalopathy, metabolic encephalopathy, vasculitis, cerebral ischaemia and paraneoplastic encephalopathy. In the rest (*n* = 23) the diagnosis was undetermined at the time of the study.

## Discussion

Among patients presenting to hospitals in Sri Lanka with a clinical syndrome of encephalitis and in whom an autoimmune aetiology was suspected as ‘possible’ by the attending neurologist, we found that NMDARE was the commonest form of AE accounting for 45.8% while LE accounted for only 4.2%. However, NMDAR-antibodies were detected only in about half of the patients with probable NMDARE while none of the common putative antibodies were detected in LE. Although there were no clinical markers that could reliably differentiate between antibody-positive and antibody-negative probable NMDARE, the EEG was found to be abnormal more frequently in antibody-negative probable NMDARE while a better outcome and fewer deaths were noted among antibody-positive probable NMDARE. Among patients who did not fulfil diagnostic criteria for ‘probable’ AE, many subsequently were recognised to have an alternative diagnosis that initially mimicked a ‘possible’ AE.

Detection of antibodies against neuronal cell-surface or synaptic proteins are an essential requisite for a definite diagnosis of AE. However, since these antibody assays are not widely available, particularly in resource poor settings, and since false negative results are known to occur [11, 12], the syndromic approach introduced in 2016 [10] based on neurological assessment and conventional tests that are widely accessible has proven to be useful as also shown in our study. The inability to detect autoantibodies does not rule out a diagnosis of AE, particularly if consistent with a syndromic diagnosis of AE and logical differential diagnosis to exclude an alternative aetiology. Antibody-negative AE is a recognised entity [6, 13, 14], usually based on response to immunotherapy in clinically suggestive patients and is attributed to lack of sensitivity of the assay used, low titres, timing of the assays and the possibility of unrecognised pathogenic antibodies that are not yet tested. Indeed, more than half of the probable NMDARE patients and all the LE patients in our study were antibody negative. However, the excess antibody negativity in our study may have also been contributed by a less stringent clinical ascertainment by the on-site neurologists and a relatively lesser sensitivity of commercial, fixed cell-based assays compared to live cell-based assays [15]. It is tempting to postulate that the lower assay sensitivity may have been related to lower AE antibody titres among South Asians given similar results in an Indian population [14], but this has not been verified. Unlike NMDARE with a single antibody target, LE with many antibody targets and more being recognised, is more likely to be diagnosed in the absence of detectable antibodies. Immunohistochemistry on murine brain section and live cell-based assays have been shown to improve antibody detection [15, 16], but these methods were not available to us. Nonetheless, 67.7% of our AE patients showed a good response (mRS < 3) to immunotherapy irrespective of their antibody status. Furthermore, most patients in our study improved with first line immunotherapy while only about one tenth required second line immunotherapy with rituximab to which the response rate was over 80% consistent with previous observations [3]. Interestingly, our data seem to suggest a better outcome among patients with detectable antibodies than those without.

Abnormal (psychiatric) behaviour or cognitive dysfunction, seizures and movement disorders were the commonest clinical manifestations of probable NMDARE among the South Asian patients in our study, which was not different to the clinical manifestations of NMDARE described in Caucasian populations [2, 17]. Similar to other populations, probable NMDARE in our study was common among young patients (two thirds below the age of 18 years) with a female preponderance while LE was common among older patients albeit with a female preponderance.

Since most patients initially manifest psychiatric features while movement disorders and seizures often occur at a later stage [4] and since the detection of autoantibodies in psychiatric illness does not necessarily mean an autoimmune aetiology [17], the finding of abnormalities on EEG in the majority of probable NMDARE patients in our study provides important evidence for greater reliance on EEG to classify the aetiology of psychiatric manifestations. Moreover, a significantly higher rate of EEG abnormalities among antibody-negative than antibody-positive probable NMDARE patients in our study further enhances the diagnostic utility of the EEG in the absence of a definite disease marker.

Although around half of the patients were first classified as ‘possible’, this did not confer any harm to the patient, but increased the diagnostic yield of ‘probable’ AE. Early treatment remains the cornerstone of a good outcome of AE [3], and in this context, the benefit of early diagnosis outweighs the disadvantage of misclassification during the early phase of the illness. Considering the response to immunotherapy in the diagnostic criteria may reduce the initial misclassification, but this is not practical because this information is not available at the time of symptom onset or early clinical evaluation. Furthermore, the lack of response to first line immunotherapy does not necessarily rule out AE and conceivably we may have missed some patients with AE in our study.

Less than 200 cases of AE has been reported from South Asia from among a cumulative population of 2 billion living in eight countries. This reflects a scarcity of published data rather than the occurrence of disease among the populations of this region. Our study represents the largest series of patients in a single study from South Asia. More importantly, this study enabled the establishment of cell-based assays at the Medical Research Institute in Colombo and provided the necessary evidence to convince the Ministry of Health of Sri Lanka to provide budgetary allocation to continue provision of AE diagnostic assays to the National Health Service which is free of charge to all citizens in Sri Lanka.

There are limitations to our study. Patients were recruited from only government hospitals with access to a neurologist, thus underrepresenting the true AE population. All patients and their investigations could not be personally examined by the research team since they were resident in many districts in the country. One year follow up data was limited to only those diagnosed with AE, and even among them, some were lost to follow up. Not all patients had access to MRI for brain imaging, which may have influenced the initial classification of AE. An infectious aetiology may have been more commonly identified had more specialised microbiological investigations been utilised in this study than only the routine investigations that were used. Indeed, in 16% of patients with possible AE, the aetiology remained undetermined.

## Conclusions

Our study adds to fill the hiatus of evidence of the frequency, clinical manifestations and prevalence of autoantibodies against neuronal cell-surface/synaptic proteins in AE among South Asians, illustrates the real world evaluation of the syndromic approach in establishing a diagnosis when antibodies are unavailable and suggests a greater reliance on EEG as a marker to evaluate for an autoimmune basis for psychiatric symptoms.

## Data Availability

All relevant data generated or analysed during this study are included in this published article.

## Abbreviations

Ab: Antibody
AE: Autoimmune encephalitis
AMPAR: α-amino-3-hydroxy-5-methyl-4-isoxazolepropionic acid receptor
CASPR2: Contactin-associated protein-like 2
CBA: Cell-based assay
CSF: Cerebrospinal fluid
CT: Computerised tomography
EEG: Electroencephalogram
GABAB: γ-aminobutyric acid B
HEK: Human embryonic kidney
LE: Limbic encephalitis
LGI1: Leucine-rich glioma-inactivated protein 1
MRI: Magnetic resonance imaging
NMDAR: N-methyl D-aspartate receptor
NMDARE: NMDAR-antibody encephalitis
